# Distribution of CpG Motifs in Upstream Gene Domains in a Reef Coral and Sea Anemone: Implications for Epigenetics in Cnidarians

**DOI:** 10.1371/journal.pone.0150840

**Published:** 2016-03-07

**Authors:** Adam G. Marsh, Kenneth D. Hoadley, Mark E. Warner

**Affiliations:** 1 Marine Biosciences, School of Marine Science and Policy, University of Delaware, Lewes, DE, United States of America; 2 Center for Bioinformatics and Computational Biology/Delaware Biotechnology Institute/University of Delaware, Newark, DE, United States of America; Northeastern University, UNITED STATES

## Abstract

Coral reefs are under assault from stressors including global warming, ocean acidification, and urbanization. Knowing how these factors impact the future fate of reefs requires delineating stress responses across ecological, organismal and cellular scales. Recent advances in coral reef biology have integrated molecular processes with ecological fitness and have identified putative suites of temperature acclimation genes in a Scleractinian coral *Acropora hyacinthus*. We wondered what unique characteristics of these genes determined their coordinate expression in response to temperature acclimation, and whether or not other corals and cnidarians would likewise possess these features. Here, we focus on cytosine methylation as an epigenetic DNA modification that is responsive to environmental stressors. We identify common conserved patterns of cytosine-guanosine dinucleotide (CpG) motif frequencies in upstream promoter domains of different functional gene groups in two cnidarian genomes: a coral (*Acropora digitifera*) and an anemone (*Nematostella vectensis*). Our analyses show that CpG motif frequencies are prominent in the promoter domains of functional genes associated with environmental adaptation, particularly those identified in *A. hyacinthus*. Densities of CpG sites in upstream promoter domains near the transcriptional start site (TSS) are 1.38x higher than genomic background levels upstream of -2000 bp from the TSS. The increase in CpG usage suggests selection to allow for DNA methylation events to occur more frequently within 1 kb of the TSS. In addition, observed shifts in CpG densities among functional groups of genes suggests a potential role for epigenetic DNA methylation within promoter domains to impact functional gene expression responses in *A. digitifera* and *N. vectensis*. Identifying promoter epigenetic sequence motifs among genes within specific functional groups establishes an approach to describe integrated cellular responses to environmental stress in reef corals and potential roles of epigenetics on survival and fitness in the face of global climate change.

## Introduction

Past climate cycles have impacted ocean temperatures and chemistry, yet fossils show scleractinian corals have been persistently present [1]. For example, ocean temperatures may have risen during past climate cycles by a net of 5°C and exceeded 30°C during the Paleocene-Eocene Thermal Maximum (PETM; ca. 55.8 Ma), however, reef carbonate production has been maintained across geologic records [2] and reef growth has proceeded unabated through centennial to millennial warming episodes following the Last Glacial Maximum (ca. 20 ka) [3]. Despite environmental impacts of recent global climate change on corals, some species potentially have the capacity for rapid acclimatization to various environmental stressors. [4–9]. Notwithstanding substantial differences in the rate of climate change for the Anthropocene as well as other anthropogenically driven environmental stressors, given that corals have successfully acclimatized or adapted to previous climatic changes, we ask if genomic level mechanisms active during such past events can be identified now in the genomes of modern corals, specifically, and cnidarians in general?

Open access to coral genomic databases [10], along with recent improvements to gene annotations allow for meaningful bioinformatic analyses of DNA nucleotide sequence patterns and motif usage in upstream gene domains [11]. Methylation of cytosine nucleotides is a prominent epigenetic mechanism with possible roles in gene expression regulation during environmental acclimatization and/or adaptation [12]. Organisms with known active methylation systems possess distinct patterns in cytosine-guanosine dinucleotide densities (CpG motifs) in the upstream promoter domains of genes in animal genomes [13–15]. Some invertebrates show prominent methylation activities, particularly in the context of development and environmental stress [16–20] and particular recent interest has focused on corals [21, 22].

Examination of CpG composition of gene bodies in relation to differential gene expression under environmental stress was compiled for the reef coral *Acropora millepora* [21]. Here, Dixon *et al*. [21] delineated genes into two groups based on CpG motif densities (high and low) and were able to show that the “low” group (i.e., genes with a weak potential for cytosine methylation) were more likely to be differentially expressed in response to stress than genes with a high potential for heavy methylation. Here, a putative link between DNA methylation and differential gene expression in *A. millepora* provides support for a role of epigenetic mechanisms in acclimatization.

Similarly, a recent study has surveyed gene expression profiles in six reef building corals in response to stress and also correlated gene body CpG composition with differential expression [22]. In three coral species exposed to temperature and ocean acidification stressors, genes with low CpG motif densities are correlated to gene transcriptional plasticity suggesting that hypomethylation may be important for maintaining responsiveness in cellular transcript pools to environmental changes. Overall, this work indicates the possible significance of epigenetic shifts in gene body methylation at CpG motifs in coral genomes.

In another recent study, coral acclimatization to temperature at a field site was described in an elegant reciprocal transplant study between tidal pools with different thermal histories [7]. Specifically this analysis identified a set of approximately 70 genes in the coral *Acropora hyacinthus* that were critically involved in acclimation to a more thermally tolerant phenotype. Given the linkages between gene expression patterns and gene body methylation described above ([21, 22]) and reported in oysters [18], we wondered if patterns of CpG motif usage in the promoter domains of genes responsive to environmental stressors might be conserved and if so would they be present in other cnidarian genomes.

In this paper, we describe the distribution of CpG dinucleotide motifs in the 5’ upstream regions of genes in two cnidarians: the reef coral *Acropora digitifera* and the sea anemone *Nematostella vectensis*. This is in contrast to the recent studies reporting CpG usage in whole gene bodies (i.e., including exons and introns) in reef corals [21, 22]. Our approach departs from previous studies by assessing CpG densities specifically across upstream promoter domains [14, 15, 23]. We also establish two comparative controls and add functional gene groupings to assess the following null hypotheses: In the absence of functional selection for CpG-site methylation, CpG densities: a) should be determined by the random distribution of C and G nucleotides within a genome, and b) should be equivalent to the mirror-image GpC dinucleotide density distributions.

## Materials and Methods

Overall, our approach used density of CpG sites in running average windows to identify spatial increases and decreases in CpG composition. We know in eukaryotes that %CpG composition rises with proximity to the transcriptional start site (TSS) [14, 15, 23] but our approach was aimed at capturing the spatial structure or signal of CpG peaks and valleys shared among genes within functional groupings. This bioinformatic approach is similar to the analyses conducted by Dixon *et al*. [21] and by Dimond and Roberts [22], both of which review background material on CpG density profiling. However, in this paper, we are exclusively characterizing the 3 kb upstream promoter domains rather than full gene bodies. Epigenetic mechanisms of gene regulation via promoter sequence elements have been well characterized in vertebrate molecular genetics since the 1990’s [24, 25].

Genomes of *A. digitifera* and *N. vectensis* were obtained from publicly available resources providing annotated genes [10]. Overall, there were 23,677 gene models for *A. digitifera* with 18,569 of those having 3 kb upstream sequences. For *N. vectensis*, there were 27,273 gene models with 16,610 of those having 3 kb upstream sequences. We used defined transcriptional start sites (TSS) as reference positions to isolate -3000 bp upstream and +500 bp downstream from each TSS. Genome annotations for *A. digitifera*, including TSS positions, were obtained from Shinzato *et al*. [10] and are publicly available through a resource portal maintained by the Okinawa Institute of Science and Technology (marinegenomics.oist.jp). Annotations for *N. vectensis* are also publicly available and were downloaded through the Joint Genome Institute’s (JGI) genome portal (genome.jgi.doe.gov). An important assumption underlying any informatic study such as this is that the predicted gene annotations and the TSS designations are biologically accurate. Although these TSS positions have not been experimentally validated for cnidarian genes, the raw CpG frequency profiles we report for these gene sets are identical in spatial patterning around the TSS as has been found in other animal genomes that have been analyzed for CpG motif frequencies [13, 14]. Our study is aligned with current approaches for handling *in silico* annotated gene sets [26].

Randomized control gene sequences were generated in proportion to known nucleotide frequencies for each cnidarian genome. Each gene model was given a 3 kb upstream domain and a 500 bp first exon of random codons drawn from a codon usage table derived from annotated protein coding genes for each species. The mirror-image GpC control was compiled using the same density analysis as the CpG dinucleotide pattern, but with the nucleotide sequence reversed. A standard weighted moving average with a 51 nucleotide window was used to calculate the density pattern as a function of distance from the transcriptional start site (TSS) between -3000 bp upstream to +500 bp downstream into the first exon. Codon usage within the 500 bp first exon of each randomized gene therefore mirrors codon usage within the corresponding species.

CpG and mirror-image control GpC motif usage were calculated as a standard weighted moving average with a 51 nucleotide window for each gene fragment (from -3000 bp upstream of TSS to +500 bp downstream). For KEGG functional analyses, a list of transcript models for each species with corresponding KEGG Class information were obtained using the Zoophyte database, which lists 19,000 *A. digitifera* sequences with KEGG annotations (bioserv7.bioinfo.pbf.hr) [11]. Aligning these gene models of Dunlap *et al*. [11] to the models we obtained for *A. digitifera* and *N. vectensis* (BLAST, *e* <10^-8^), yielded 14,162 KEGG classified genes for the former and 10,463 for the latter. These functional classifications contained the KEGG gene identifiers that were used to group genes into higher order functional processes. For all statistical comparisons, only genes that were uniquely assigned a single functional KEGG class were used. These gene counts are presented in Table 1. For both species, homologs of the environmental adaptation genes presented in Palumbi *et al*. [7] were identified using BLAST alignment (*e* <10^-8^ cut off). For *A. digitifera* this produced a set of 122 genes and for *N. vectensis*, a set of 72 genes.

**Table 1:** KEGG Gene Groups. KEGG Class information was obtained using the Zoophyte database for 19,000 *A. digitifera* gene sequences [11]. Class groups were then filtered for genes only having a single unique class grouping. Numbers of genes in these sets for both genomes are shown.

	*Acropora digitifera*		*Nematostella vectensis*	
KEGG CLASS	Total	Unique	Total	Unique
Metabolism	4,112	1,950	4,185	1,400
Environmental Information Processing	3,060	2,106	2,757	1,880
Genetic Information Processing	2,196	1,458	1,617	1,208
Cellular Processes	3,098	1,442	1,934	927
Organismal Processes	5,573	1,237	3,964	822

### Statistical Analysis

To test for significant differences across KEGG class categories, gene promoter regions were divided into four regions of nucleotide length and CpG motif frequencies were calculated for each section. A Kruskal-Wallace test with Bonferonni correction was then performed on each section. If significant differences were observed, a Dunn’s test was utilized to compare rank sums between KEGG class categories. For comparisons between the subset of genes from Palumbi *et al*. [7] and the full gene set within each species, genes sets were similarly analyzed but with a Wilcoxon rank sum test to identify significant differences. All statistical analyses were performed in R, utilizing the ‘PMCMR’ package.

### Resources

#### Publicly Available Genomes

Genomic gene sequence data for *A. digitifera* were sourced from:

  http://marinegenomics.oist.jp/genomes/downloads?project_id=3

Genomic gene sequence data for *N. vectensis* were sourced from:

  http://genome.jgi.doe.gov/Nemve1/Nemve1.info.html

#### Available Project Code

PERL Scripts and gene data sets used in this paper are available from K.H at https://github.com/khoadley. FASTA files for both cnidarian data sets have sequence headers that include the corresponding gene annotation along with the KEGG KO number if available. Genes utilized in the Temperature Acclimation group are additionally annotated with an identifier of the corresponding gene homolog reported in Palumbi *et al*. [7].

## Results and Discussion

**Fig 1:**
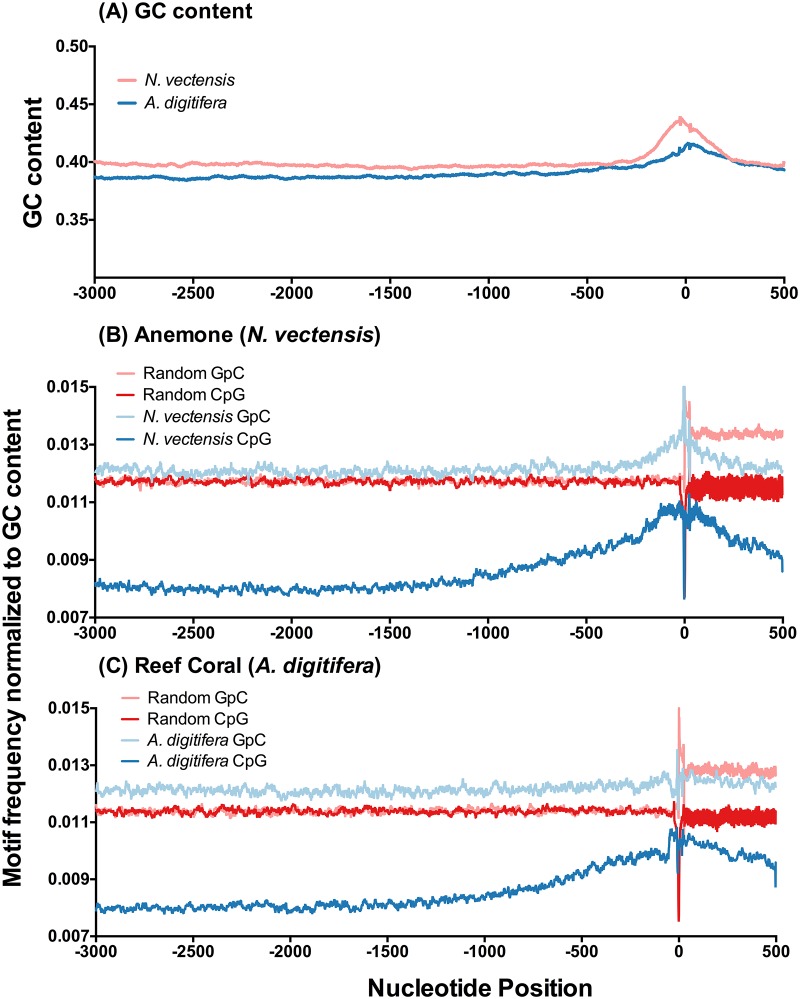
Total CpG Profiles. Mean nucleotide usage in annotated genes across upstream domains plotted as running averages. A: Fractional G+C content for the two cnidarians. B: The prevalence of CpG and GpC motifs across upstream domains from -3000 bp to +500 bp around TSS (x = 0) was calculated as a 51 bp moving average that was normalized to G+C content (shown in A) for the anemone *N. vectensis* (n = 16,610 genes). C: CpG and GpC motif usage for the reef coral *A. digitifera* (n = 18,569 genes). In both B and C, fractional representation of these motifs are shown for a set of 20,000 randomized control gene models based on genome sequence composition of each species.

For both cnidarian genomes, G+C fractional content in upstream domains is shown in Fig 1A as a 51 bp moving average calculated for each defined gene and then averaged over all genes within each respective genome. The coral *A. digitifera* shows a slight rise in G+C levels across this region, with a steep change between -250 bp and transcriptional start sites (TSS). The anemone *N. vectensis* shows equivalent G+C levels across upstream domains with a large rise between -250 bp and TSS. Spatial changes in G+C distributions (relative to the TSS) are characteristic of metazoan genomes [13, 14] with upstream sequence changes resulting from increases in G and C nucleotide retention (or persistence) near the TSS where promoters, enhancers and repressors are more likely to actively influence gene expression [24, 25].

In these genomes, CpG motif frequencies increase across the upstream domain from -2000 bp to TSS (ca. 1.38x; Fig 1B and 1C). This increase is not reflected in mirror-image GpC motif frequencies, indicating a selection bias for CpG motif usage independent of G+C content. In comparison to control randomized gene models (n = 20,000), observed CpG motif usage in both cnidarians shows spatial patterns highly differentiated from expected C and G pairing in a random, null selection model. In contrast, the mirror motif GpC shows usage patterns equivalent to a random model of G and C pairings in upstream domains. Hence, in comparing usage patterns of CpG vs. GpC, there is an apparent directional selection for the depletion of CpG motifs relative to the levels of GpC occurrence in promoter domains. These trends shift at the TSS where codon usage frequencies in the first exon impact nucleotide composition.

CpG motif frequencies in upstream domains show lower representation than a random null selection expectation. This spatial pattern of depletion is noted in metazoans [23] and may result from molecular processes involving differential 5’-methyl-cytosine to thymidine transition mutation frequencies. Over evolutionary time-scales, these lower CpG motif frequencies persist. Overall reductions in CpG motifs suggest functional limitations on motif abundance, especially when the reverse GpC motif shows no corresponding depletion in usage. Directional selection towards under-representation of CpG motifs in upstream domains provides evidence that CpG motif frequencies are functionally important and that limitations on frequency of their usage exist at some level of fitness. Given correlations recently established between gene body CpG frequencies and gene expression patterns in corals [21, 22], and extrapolations to roles of hyper- vs. hypomethylation states, it is highly likely that cytosine methylation plays some role in gene regulation events in invertebrates in general [27], and here in these two cnidarians specifically.

**Fig 2:**
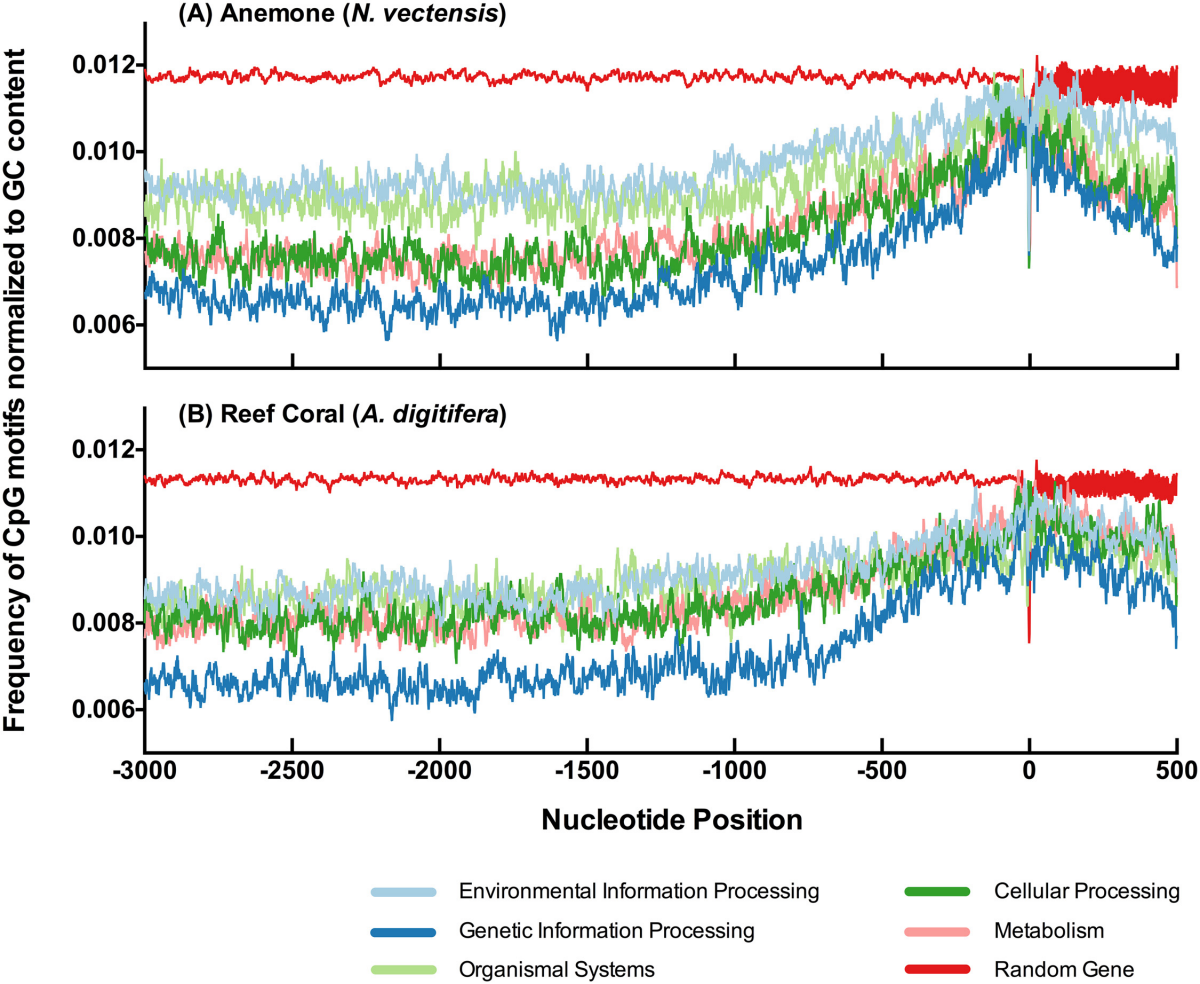
Functional Gene Groupings. CpG motif frequencies in upstream domains of gene sets for KEGG functional classes. There are five functional classes: Environmental Information Processing, Genomic Information Processing, Organismal Systems, Cellular Processing and Metabolism. A: gene group profiles for anemone *N. vectensis*. B: gene group profiles for reef coral *A. digitifera*. Respective randomized gene sets are plotted for reference. Numbers of genes in each group are indicated in Table 1.

We then profiled CpG motif frequencies among functional gene categories using KEGG class designations [28]. By dividing annotated genes into specific functional class groups and repeating analyses as in Fig 1, differences in CpG motif frequencies among gene groups is evident. Overall, some functional groups reveal linear patterns in signal peaks and valleys in CpG densities across upstream domains (Fig 2). Within the Genetic Information Processing (GIP) group, CpG usage was lower and exhibited prominent spatial conservation in the location of peaks and valleys. The Environmental Information Processing (EIP) group and Organismal Systems (OS) group generally show higher CpG motif frequencies far upstream then converge on equivalent values at the TSS. Methylation in promoter domains may control quiescent genes that are sporadically needed (e.g. EIP), while constitutively expressed genes can be predominantly unmethylated across their promoters (e.g. GIP) [27]. The periodic high and low signal features in these profiles are suggestive of spatial CpG site usage conserved across genes related by function. More importantly, many genes in the EIP group are putatively involved in cellular responses to environmental change and may be associated with cellular acclimitization processes.

Interestingly, this result of higher CpG motif frequencies in the upstream promoter domains of environmental response-linked genes (EIP) is different than the pattern found with CpG motif frequencies in gene bodies [21, 22]. In those studies, gene bodies of transcripts that were upregulated in response to environmental stressors showed lower CpG motif frequencies which in both cases lead to the authors’ conclusion that hypo-methylation (low methylation potential via low CpG motif frequencies) in gene bodies was important for genes involved with environmental acclimation responses. Given what is known about vertebrate promoter CpG methylation [24, 25] and what we are beginning to uncover in invertebrate gene promoters [27], there is a potentially interesting difference here in molecular function between promoter domains and gene bodies in CpG motif usage.

**Fig 3:**
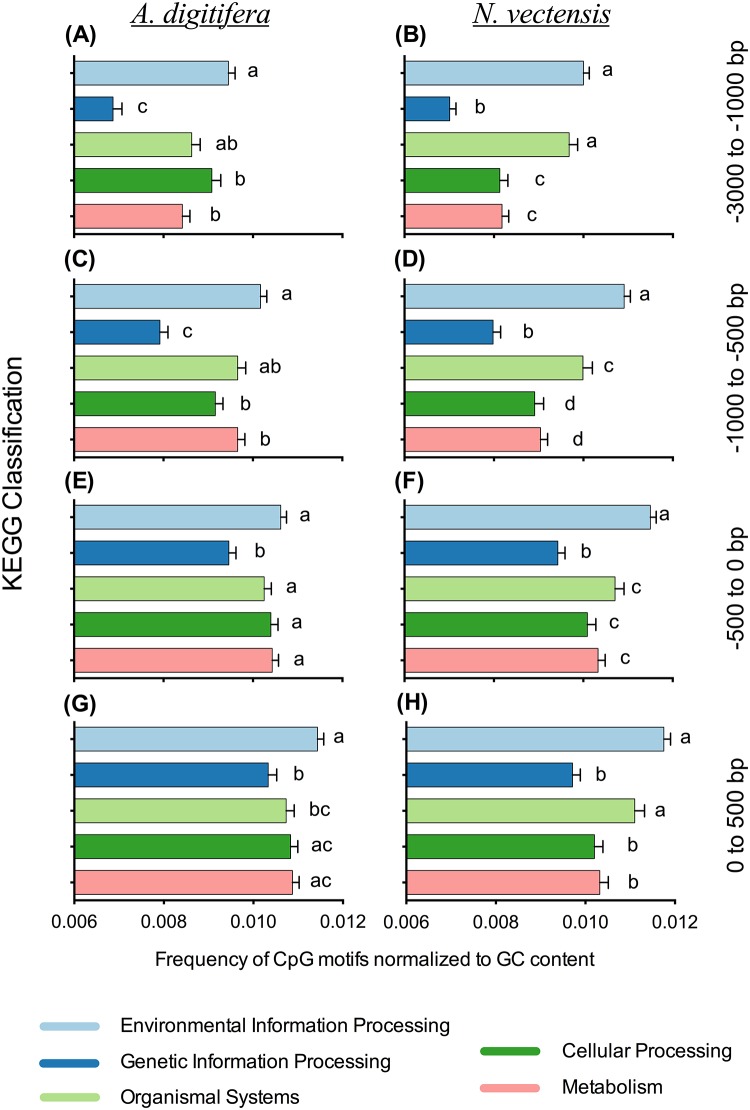
CpG Region Frequencies in Functional Gene Groupings. The 3 kb upstream promoter domains were divided into four position regions: +500 to 0, 0 to -500, -500 to -1000 and -1000 to -3000. CpG motif frequencies were calculated across each region for unique genes in five KEGG functional classes. Plots for both cnidarian gene sets are shown as mean frequencies ± SE: *A. digitifera*—A, C, E, G; *N. vectensis*—B, D, F, H. Significantly different groups (*α* = 0.05) within a region for each cnidarian were determined by a Dunn’s rank test.

We assessed if CpG motif frequencies in different regions of the 3 kb upstream domain differ across functional categories. Grouping unique genes by KEGG Class (Table 1) and then calculating CpG motif frequencies across four different gene regions, we were able to identify statistically different spatial patterns of CpG usage among different functional classes of genes (Fig 3). The most consistent significant difference in both cnidarians across all regions is a high CpG motif frequency in EIP genes and low CpG frequency in GIP genes, with the difference in frequencies between these two groups increasing with distance from TSS (0 position). The other three functional groups (Organismal Systems, Cellular Processes and Metabolism) are intermediate between EIP and GIP frequencies and evidence a gradual decline away from TSS. The statistical comparisons in Fig 3 indicate that CpG motif frequencies in different upstream regions may be conserved among genes within discrete functional groupings and could potentially be indicative of common regulatory mechanisms for coordinated transcription.

**Fig 4:**
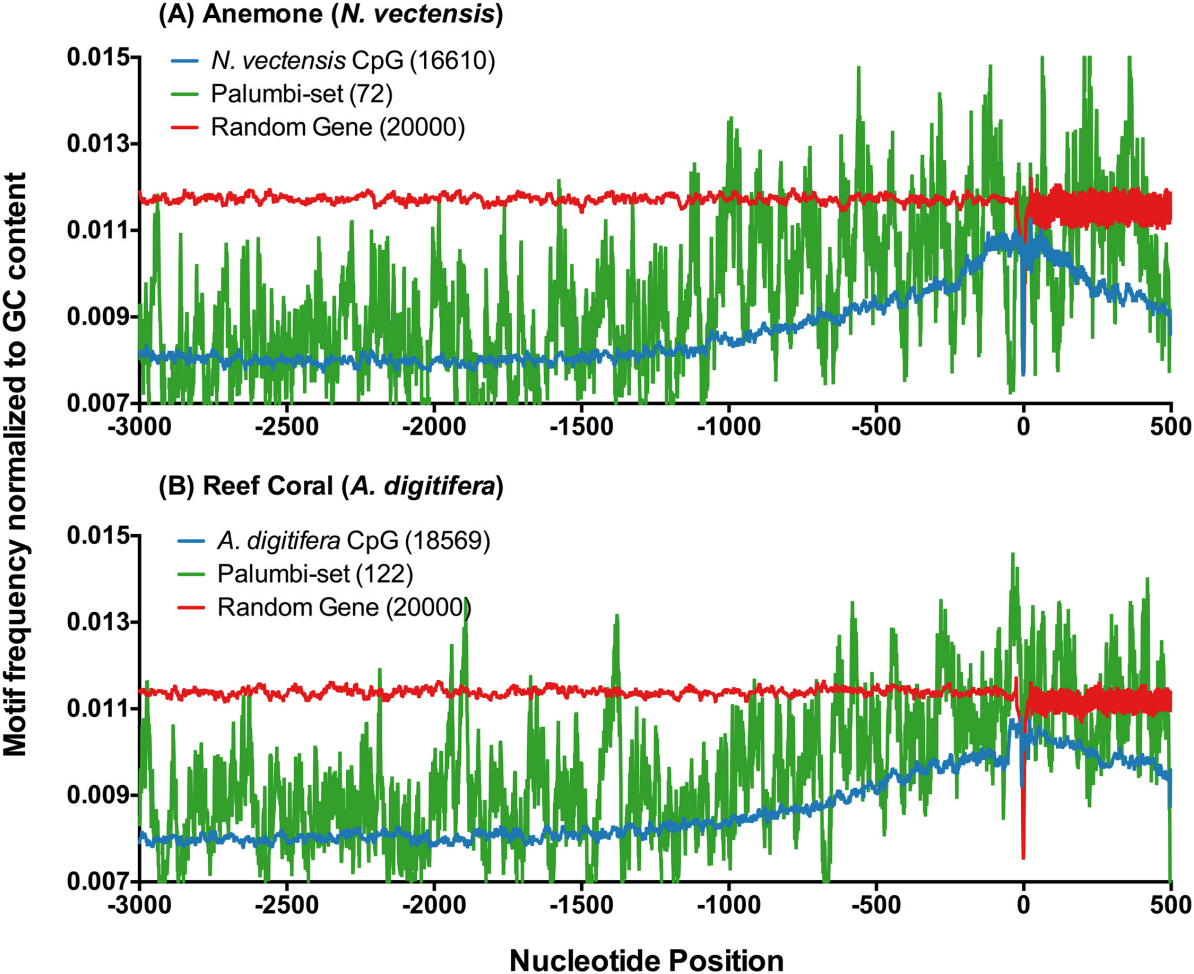
Genes Active in Environmental Acclimatization. CpG motifs across upstream domains from -3000 bp to +500 bp around TSS. Genes represented by the green trace are homologs of genes from reef coral *A. hyacinthus* showing high acclimatization potential as identified by Palumbi *et al*. [7]. The whole genome (blue) and randomized genome (red) are included for reference.

In the coral *Acropora hyacinthus* an acclimation response to temperature has been recently described in an elegant study [7]. Specifically this study identified a set of genes that were involved in temperature acclimation to a more thermally tolerant phenotype between field sites with different local temperature regimes. Using this functional group of temperature acclimation genes in a coral, we analyzed CpG motf frequencies in their 3 kb upstream domains. In Fig 4, CpG spatial patterns for these genes in *A. digitfera* and the more distantly related *N. vectensis* reveal common periodic peaks and valleys with large amplitudes. Compared to CpG patterns of the EIP group in Fig 2, these temperature acclimation genes (Fig 4) show pronounced conservation in spatial patterns (periodic peaks and valleys) of CpG occurrence.

**Fig 5:**
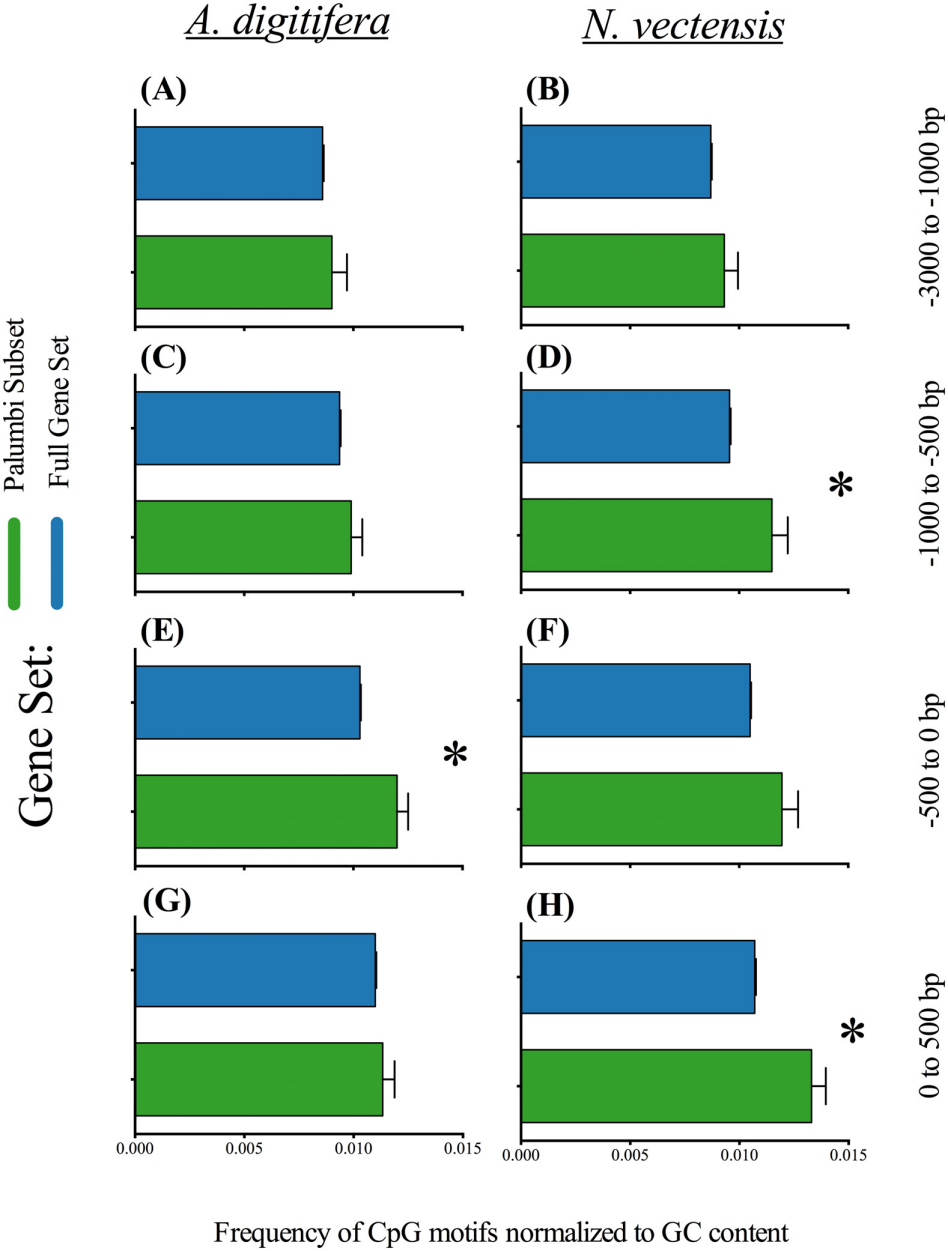
CpG Motif Frequencies in the Temperature Acclimation Gene Set. Upstream promoter domains were divided into four position regions: +500 to 0, 0 to -500, -500 to -1000 and -1000 to -3000. CpG motif frequencies were calculated across each region for two gene sets: the set of temperature acclimation genes from Palumbi *et al*. [7] and the set of remaining genes. Plots for both cnidarian gene sets are shown as mean frequencies ± SE: *A. digitifera*—A, C, E, G; *N. vectensis*—B, D, F, H. Significantly different groups (*α* = 0.05) within a region for each cnidarian were determined by Wilcoxon’s rank sum test. Significant differences are indicated by an asterisk.

We compared CpG motif frequencies in different upstream regions of this specific acclimation gene set to the full gene set for each cnidarian (Fig 5). In *A. digitfera* the -500 to TSS region shows significantly greater CpG motif frequencies, while in *N. vectensis* the TSS to +500 region and the -1000 to -500 region CpG motif frequencies are significantly higher. Thus, within these temperature acclimation genes [7] there appears to be equivalent sequence blocks of CpG usage at discrete intervals relative to TSS. This conservation suggests methylation states of discrete CpG sites could play a role in environmental acclimatization (EIP, Figs 2 and 3), and particularly acclimatization to temperature stress (Fig 5).

The gene promoter profiling work was stimulated by an interest in understanding if epigenetic cytosine methylation could be an important molecular mechanism by which cnidarians could acclimate to changing environments. Two recent studies have been pivotal to shaping this approach: 1) the identification of a suite of cnidarian genes in *Acropora hyacinthus* that were experimentally shown to positively contribute to a genetic-level temperature acclimation response [7]; and 2) the identification of a linkage between gene body methylation and gene expression events [21, 29]. Using a defined group of genes presented in the former and the focus on gene body methylation mechanisms utilized in the latter, we’ve pursued a description of differential CpG motif frequencies in upstream gene promoter regions in two other cnidarians, one a congener and one a more distantly related anemone. In these cnidarians, we uncovered a high-degree of differential CpG usage in gene promoter domains among functional (KEGG) gene sets.

The prevalence of CpG motif patterns in the cnidarian gene sets we examined suggests functional activities of cytosine methylation in gene promoter domains. Localizing these differential usage patterns to gene groups associated with environmental acclimatization shows a nucleotide sequence pattern for potential epigenetic regulation of gene expression. Keeping in mind the significant CpG profiles in whole gene bodies that have been correlated with differential gene expression in corals [21, 22], it is likely that DNA methylation of cytosine is an important molecular mechanism involved in coordinating cellular responses to environmental stress. Although this statement is still a working hypothesis formed by our bioinformatics approach, it is testable in the near future. Using quantitative, site-specific, DNA methylation profiling [17, 18], we will be able to assess contributions of CpG methylation shifts to altered cellular and/or organismal physiological phenotypes.

Although tropical coral reefs are delicate ecosystems in terms of existing in a complicated balance of chemical, biological and ecological interactions, and changes in the Anthropocene are happening on accelerated time scales, there is evidence indicating that some coral species may be more resilient than thought, and capable of near-term acclimatization in the face of environmental change [4, 5, 7, 21, 22, 30]. Over generational time-scales, there is also potential for corals to adapt to changing climate regimes [31]. The mechanism by which some tropical corals may be able to survive and persist global climate change could involve an integrated molecular response involving epigenetics (both histone and DNA modifications), nucleotide sequence variants (single nucleotide polymorphisms [SNP] and insertion or deletion mutations), transposable element activities, micro-RNA pools, transcriptome, proteome, and metabolome processes. Most of the current work with corals has focused on characterizing differences in transcriptomes. However, the SNP profiling work of Bay and Palumbi [31] describes a potential at the level of population genetics for coral allelic diversity to increase fitness of thermal tolerant phenotypes relatively quickly under temperature selection. Attention is now turning to the role of epigenetics in altering gene expression activities. As more molecular response data is collected across different molecular processes, an integrated “omics” picture of coral acclimatization and adaptation will emerge.

A key extension of the results of the present work is the recognition that genes important for temperature acclimation may be revealed by their promoter regulatory structure of CpG spatial patterns. It is possible that there is a fingerprint of CpG usage that could be used to identify other genes coordinately regulated during temperature acclimatization. Genes that are important for acclimation do not necessarily need to be highly up-regulated (in terms of 2x transcription rates) to participate in acclimation processes, they just need to be expressed or active. Also, transcript splicing variants or post-translational modifications impacting protein half-life or intracellular trafficking changes could decouple the absolute levels of a gene transcript from its situational fitness value to cellular biochemistry/physiology. Thus, there may be ‘cryptic’ acclimation genes that are not discoverable solely via transcriptome profiling, but which may be identified by their CpG methylation dynamics. Understanding the linkage between CpG usage patterns and cytosine methylation dynamics could open new research approaches to identifying functionally relevant gene sets in natural populations that are linked to environmental acclimation and adaptation through epigenetic processes.

We have characterized promoter structure for sequence orthologs of the 70+ *Acropora hyacinthus* temperature acclimation genes in two other cnidarians: *Acropora digitifera* and *Nematostella vectensis*. We present evidence that CpG dinucleotide motifs, which are active sites of cytosine methylation and hence under possible epigenetic regulation, are under-represented in these promoter domains. What we need to experimentally determine now is whether epigenetic cytosine methylation changes are an important dynamic mechanism for regulating temperature acclimation responses in cnidarians and whether methylation shifts are shared within a group of environmental “response” genes. The direct linkages that have been described for *Acropora hyacinthus* between environment and genes [7, 30] and CpG content in genes [21, 22] break ground for others to explore the multiple dimensions by which epigenetics may contribute to coral acclimatization responses.
